# Supplementary material for: Uncovering the heterogeneity and temporal complexity of neurodegenerative diseases with Subtype and Stage Inference
Source: Nat Commun. 2018 Oct 15;9:4273. doi: 10.1038/s41467-018-05892-0 (PMC6189176; doi:10.1038/s41467-018-05892-0)
Supplement: Supplementary file 1 — Supplementary Information [file 41467_2018_5892_MOESM1_ESM.pdf]

Supplementary Information

Uncovering the heterogeneity and temporal complexity of neurodegenerative diseases with Subtype and Stage Inference

Young et al.

Supplementary Methods

Simulations

To verify the ability of the SuStaIn algorithm to reveal subtypes and their progression patterns from heterogeneous patient snapshots we performed a stability analysis using simulated data. We simulated data drawn from the linear z-score model described in Methods: Mathematical model. We chose a set of default model parameters described subsequently, and then varied each parameter individually to establish the effect of different settings on the recovery of the subtypes and stages by the SuStaIn algorithm.

Default settings

By default we set the number of subjects J to $J = 500$, number of biomarkers I to $I = 10$, number of clusters C to $C = 3$, and biomarker covariance $\mathbf{\Sigma}$ to the identity matrix (no covariance). We simulate the SuStaIn stages from a uniform distribution, $P(t) = 1$, and hence $P(k) = \frac{1}{N+1}$. We simulate the SuStaIn subtypes using a fraction $f_c = \frac{2+(C-c)}{2C+\sum_{c=1}^{C}(C-c)}$. This means that for the default number of clusters $C = 3$ we have $f_1 = \frac{4}{9}, f_2 = \frac{3}{9}, f_3 = \frac{2}{9}$. We simulate the progression pattern for each subtype to be a linear z-score model, parameterised by a sequence of z-score events with a random monotonic ordering (see Methods: Mathematical model), fixing $z_i = (1,2,3)$ and $z_{i,\max} = 5$ for all biomarkers i.

Number of subjects, biomarkers and clusters

We vary: (i) the number of subjects J as $J = 200, J = 500$ (default), and $J = 1000$; (ii) the number of biomarkers I as $I = 5, I = 10$ (default), and $I = 15$; (iii) the number of clusters C as $C = 1, C = 3$ (default), and $C = 5$. For 5 clusters the fractions are $f_1 = \frac{6}{20}, f_2 = \frac{5}{20}, f_3 = \frac{4}{20}, f_4 = \frac{3}{20}$, and $f_5 = \frac{2}{20}$.

Biomarker covariance

To assess the effect of biomarker covariance $\mathbf{\Sigma}$ we simulated covariance matrices $\mathbf{\Sigma}$ in which each matrix had ones on the diagonal and randomly sampled (symmetric) off diagonal elements. The off diagonal elements were sampled from a uniform distribution between 0 and a maximum-value parameter, $\lambda_{\max}$. We considered the settings: $\lambda_{\max} = 0$ (default), $\lambda_{\max} = 0.25$, $\lambda_{\max} = 0.5$.

Misdiagnosis

We assessed the effect of misdiagnosis by simulating increasing proportions of outliers, f_m, such that each outlier followed a random progression pattern, i.e. a linear z-score model parameterised by a sequence of z-score events with a random monotonic ordering (see Methods: Mathematical model). We considered the settings $f_m = 0$ (default), $f_m = 0.05$, $f_m = 0.1$, $f_m = 0.15$, and $f_m = 0.2$.

Distinct and overlapping modes
SuStaIn relies on the assumption of multiple distinct progression patterns, however it is plausible that the underlying data may instead come from a spectrum of disease progression patterns. We therefore performed one final set of experiments assessing the performance of SuStaIn in the presence of a spectrum of progression patterns (overlapping modes), compared to multiple distinct progression patterns (distinct modes). For the distinct modes experiment we simulated two subtypes in which $f_1 = f_2 = \frac{1}{2}$. For the overlapping modes experiment we randomly assigned individuals a proportion with which they expressed subtype 1 vs. subtype 2 using a uniform distribution over the values 0 to 1. We then simulated each individual's data as the weighted average of their predicted data from the two subtype progression patterns, given their sampled SuStaIn stage. This generates uniform samples from a spectrum of progression patterns with two distinct extremes.

Convergence
For each of the 10 datasets per parameter setting we assessed convergence of the SuStaIn algorithm by checking that the solution reached from each of the 25 random start points (a set of linear z-score models with random monotonic orderings of z-score events, and fractions $f_1 = \cdots = f_C = \frac{1}{C}$) per dataset is (a) within a 1×10^{-3}% tolerance level of the maximum likelihood solution, and (b) within the uncertainty estimated by the MCMC (see Methods: Uncertainty estimation). We further checked that initialisation of the MCMC procedure from the ground truth solution gave the same result as the solution reached by the SuStaIn algorithm.

Error in subtype progression patterns
To evaluate the accuracy of the subtype progression patterns recovered by SuStaIn we simulated 10 datasets for each parameter setting and used SuStaIn to estimate subtypes and their progression, comparing the estimated progression patterns with the ground truth progression patterns. To enable direct comparison of the subtype progression patterns (i.e. a one to one mapping between the simulated and estimated subtypes), we fixed the number of subtypes estimated by SuStaIn to be the ground truth number of subtypes for the simulated data. We tested the ability of SuStaIn to recover the correct number of subtypes separately in an additional experiment (see Number of Subtypes). We compared the simulated and estimated subtype progression patterns (as shown in Supplementary Figures 1A, 3A, 5A, 7A, 9A, 11A) by evaluating the Kendall rank correlation between the most probable subtype progression pattern estimated by SuStaIn and the ground truth. The Kendall rank correlation has a maximum of 1 when two sequences are identical, a minimum of -1 when two sequences are the inverse of one another, and an expectation of 0 when two sequences are generated independently.

Uncertainty in subtype progression patterns
In addition to estimating the most probable subtype progression patterns, SuStaIn also estimates the uncertainty in the progression patterns through MCMC sampling. To assess the utility of the uncertainty estimates obtained from SuStaIn, we tested whether a reduction in accuracy of the progression pattern is indicated by a larger variance of the distribution of sampled progression patterns obtained from the MCMC samples. Specifically, we evaluated the distribution of the position of each biomarker event in the subtype progression pattern (as shown in Supplementary Figures 1B, 3B, 5B, 7B, 9B, 11B) from the MCMC samples provided by SuStaIn. To make this distribution comparable over different experiments where the position of the

biomarker events in the subtype progression patterns is different, we subtracted the ground truth position of each biomarker event. We additionally normalised this difference by the number of biomarker events in the subtype progression pattern so that the differences were comparable across subtype progression patterns with different numbers of biomarker events.

Error in proportion of subjects in each subtype
We used the datasets described previously to determine the mean absolute error in the fraction of subjects belonging to each subtype estimated by SuStaIn (as shown in Supplementary Figures 2A, 4A, 6A, 8A, 10A, 12A).

Uncertainty in proportion of subjects in each subtype
SuStaIn further estimates the uncertainty in the proportion of subjects belonging to each subtype through MCMC sampling. We assessed the utility of the uncertainty estimates obtained from SuStaIn by testing two properties of the distribution of the sampled fractions of subjects obtained from the MCMC samples: (A) whether a reduction in accuracy of the fraction is indicated by a larger variance of the distribution of fractions, and B) whether the distribution is centred around the ground truth fraction (i.e. the estimate is non-biased). We tested (A) and (B) by evaluating the distribution of the difference between the samples of the fraction of subjects belonging to each subtype provided by SuStaIn and the ground truth (as shown in Supplementary Figures 2B, 4B, 6B, 8B, 10B, 12B). This means that the distribution will have a mean of 0 when the MCMC samples are centred on the ground truth fraction with no bias in the estimate, with a larger variance indicating a larger uncertainty estimate.

Number of subtypes
To assess the ability of SuStaIn to recover the correct number of subtypes we simulated three datasets for each parameter setting and estimated the number of subtypes by implementing the full 10-fold cross-validation procedure and comparing the CVIC (see Methods: Cross-validation) for different subtype numbers. We then compared this estimated number of subtypes to the simulated number of subtypes. We chose to use only three datasets per parameter setting to reduce the computational load, which increases 10-fold when performing the cross-validation procedure.

Supplementary Results

Convergence

In all simulated datasets for all parameter settings we find that the SuStaIn algorithm displays good convergence: the solution reached from each random start point is typically within a 1×10^{-3}% tolerance level of the maximum likelihood solution, and within the uncertainty estimated by the MCMC (see Methods: Uncertainty estimation). Moreover, in all simulated datasets for all parameter settings we found that initialisation of the MCMC procedure from the ground truth solution gave the same result as the solution reached by the SuStaIn algorithm.

Number of subjects

Supplementary Figure 1A shows the distribution of the Kendall rank correlation between the estimated subtype progression patterns and the ground truth for different numbers of subjects: 200 subjects (comparable to the GENFI dataset), 500 subjects (the default setting, comparable to the ADNI dataset), and 1000 subjects (comparable to the combination of the ADNI 3T and 1.5T). As expected, larger subject numbers correspond to increasing accuracy of the subtype progression patterns: the average Kendall rank correlation across the 10 simulated datasets is 0.86 for 200 subjects, 0.92 for 500 subjects, and 0.95 for 1000 subjects. However, even with only 200 subjects there is a high enough Kendall rank correlation to provide a meaningful indication of the subtype progression patterns. Supplementary Figure 1B shows the distribution of the position of each event stage in the subtype progression patterns, i.e. the uncertainty in the subtype progression patterns estimated by SuStaIn, for different numbers of subjects. As expected, the uncertainty in the estimated subtype progression pattern decreases with increasing subject numbers: the distribution of the position of each biomarker event has an average standard deviation across experiments of 0.10 for 200 subjects, 0.06 for 500 subjects, and 0.05 for 1000 subjects.

Supplementary Figure 2A shows the distribution of the error in the proportion of subjects assigned to each cluster for different numbers of subjects. SuStaIn accurately estimates the proportion of individuals belonging to each cluster: the mean absolute error is 0.054 for 200 subjects (an expectation of about 10/200 subjects misassigned), 0.026 for 500 subjects, and 0.022 for 1000 subjects. Supplementary Figure 2B shows the distribution of the proportion of individuals belonging to each cluster, i.e. the uncertainty in the proportion of individuals estimated by SuStaIn, for different numbers of subjects. As expected, this uncertainty decreases as the number of subjects increases, with an average standard deviation across experiments of 0.048 for 200 subjects, 0.027 for 500 subjects, and 0.019 for 1000 subjects. The distribution has a mean of zero for the 500 and 1000 subject experiments, indicating a non-biased estimate of the proportion of individuals belonging to each subtype for large subject numbers. For 200 subjects SuStaIn slightly underestimates the proportion of individuals belonging to the largest cluster, and slightly overestimates the proportion of individuals belonging to the smallest cluster.

For 200 and 500 subjects SuStaIn estimates the correct number of clusters in all simulated datasets. For 1000 subjects, SuStaIn estimates the correct number of clusters in two out of three datasets. In one dataset of 1000 subjects SuStaIn overestimates the number of clusters, giving four rather than three clusters, however, this cluster is clearly an outlier cluster accounting for only 3% of the data and with no predominant progression pattern.

Number of biomarkers
Supplementary Figure 3A shows the distribution of the Kendall rank correlation between the estimated subtype progression patterns and the ground truth for increasing numbers of biomarkers: 5 biomarkers, 10 biomarkers (default setting) and 15 biomarkers. We find that as the number of biomarkers increases, the accuracy of the subtype progression patterns stays more or less constant, with an average Kendall rank correlation of 0.90 for 5 biomarkers, 0.92 for 10 biomarkers, and 0.91 for 15 biomarkers. Supplementary Figure 3B shows the distribution of the position of each event stage in the subtype progression patterns, i.e. the uncertainty in the subtype progression patterns estimated by SuStaIn, for different numbers of biomarkers. The uncertainty in the subtype progression patterns is larger for smaller numbers of biomarkers: the uncertainty in the position of each biomarker event has an average standard deviation of 0.09 for 5 biomarkers, 0.07 for 10 biomarkers, and 0.06 for 15 biomarkers. This is likely because the trajectories of different subgroups are more strongly defined and separated by larger numbers of biomarkers.

Supplementary Figure 4A shows the distribution of the error in the proportion of subjects assigned to each cluster for different numbers of biomarkers. SuStaIn accurately estimates the proportion of individuals belonging to each cluster: the mean absolute error is 0.053 for 5 biomarkers, 0.026 for 10 biomarkers, and 0.026 for 15 biomarkers. Supplementary Figure 4B shows the distribution of the proportion of individuals belonging to each cluster, i.e. the uncertainty in the proportion of individuals estimated by SuStaIn, for different numbers of biomarkers. As expected, this uncertainty decreases as the number of biomarkers increases, with an average standard deviation across experiments of 0.049 for 5 biomarkers, 0.027 for 10 biomarkers, and 0.024 for 15 biomarkers. The mean of the distribution is centred on zero for 10 and 15 biomarkers, indicating a non-biased estimate of the proportion of individuals belonging to each cluster. For 5 biomarkers, there is a very slight underestimation of the proportion of individuals belonging to the largest cluster, and a very slight overestimation the proportion of individuals belonging to the smallest cluster.

For 10 and 15 biomarkers SuStaIn estimates the correct number of clusters in all simulated datasets. For 5 biomarkers SuStaIn estimates the correct number of clusters in two out of three datasets. In the remaining dataset two of the simulated subtype progression patterns are, by chance, similar to one another (the two least dominant subtype progression patterns have a Kendall rank correlation of 0.77). In this dataset SuStaIn underestimates the number of clusters, giving two rather than three clusters.

Number of clusters
Supplementary Figure 5A shows the distribution of the Kendall rank correlation between the estimated subtype progression patterns and the ground truth for increasing numbers of clusters: 1 cluster, 3 clusters (default setting) and 5 clusters. With increasing numbers of clusters the accuracy of the subtype progression patterns reduces, as there are fewer subjects per subtype. The average Kendall rank correlation is 0.97 for 1 cluster, 0.92 for 3 clusters and 0.87 for 5 clusters, still providing a good estimate of the subtype progression pattern for 5 clusters. Supplementary Figure 5B shows the distribution of the position of each event stage in the subtype progression patterns, i.e. the uncertainty in the subtype progression patterns estimated by SuStaIn, for different numbers of clusters. As expected, the uncertainty increases as the number of clusters increases, with an average standard

deviation across simulated datasets of 0.03 for 1 cluster, 0.07 for 3 clusters, and 0.10 for 5 clusters. The uncertainty in the subtype progression pattern is largest for the least dominant cluster, which has the smallest number of subjects.

Supplementary Figure 6A shows the distribution of the error in the proportion of subjects assigned to each cluster for different numbers of clusters: the mean absolute error is 0 for 1 cluster (the fraction is always 1 for 1 cluster), 0.026 for 3 clusters, and 0.030 for 5 clusters. Supplementary Figure 6B shows the distribution of the proportion of individuals belonging to each cluster, i.e. the uncertainty in the proportion of individuals estimated by SuStaIn, for different numbers of clusters. As expected, this uncertainty increases as the number of clusters increases, with an average standard deviation across experiments of 0 for 1 cluster, 0.027 for 3 clusters, and 0.027 for 5 clusters. The distribution is centred round zero, i.e. the estimate of the proportion of individuals belonging to each cluster is unbiased for varying numbers of clusters.

For varying numbers of clusters SuStaIn estimates the correct number of clusters in all simulated datasets for 1, 3 and 5 clusters.

Biomarker covariance

Supplementary Figure 7A shows the distribution of the Kendall rank correlation between the estimated subtype progression patterns and the ground truth for increasing amounts of biomarker covariance λ_{max}. As the covariance level increases the accuracy of the subtype progression pattern reduces very slightly: the average Kendall rank correlation is 0.92 for $\lambda_{max} = 0$ (default setting), 0.91 for $\lambda_{max} = 0.25$, and 0.89 for $\lambda_{max} = 0.5$. Supplementary Figure 7B shows the distribution of the position of each event stage in the subtype progression patterns, i.e. the uncertainty in the subtype progression patterns estimated by SuStaIn, for different levels of biomarker covariance. This uncertainty increases slightly with larger biomarker covariance, with an average standard deviation across simulated datasets of 0.07 for $\lambda_{max} = 0$ (default setting), 007 for $\lambda_{max} = 0.25$, and 0.08 for $\lambda_{max} = 0.5$.

Supplementary Figure 8A shows the distribution of the error in the proportion of subjects assigned to each cluster for different amounts of biomarker covariance λ_{max}: the mean absolute error is 0.026 for $\lambda_{max} = 0$, 0.029 for $\lambda_{max} = 0.25$, and 0.037 for $\lambda_{max} = 0.5$. Supplementary Figure 8B shows the distribution of the proportion of individuals belonging to each cluster, i.e. the uncertainty in the proportion of individuals estimated by SuStaIn, for different amounts of biomarker covariance. As expected, this uncertainty increases slightly as the covariance level increases, with an average standard deviation across experiments of 0.027 for $\lambda_{max} = 0$, 0.027 for $\lambda_{max} = 0.25$, and 0.028 for $\lambda_{max} = 0.5$. The distribution is centred round zero, i.e. the estimate of the proportion of individuals belonging to each cluster is unbiased for all covariance levels.

For varying biomarker covariance levels λ_{max} SuStaIn estimates the correct number of clusters in all simulated datasets for for $\lambda_{max} = 0$, $\lambda_{max} = 0.25$ and $\lambda_{max} = 0.5$.

Misdiagnosis
Supplementary Figure 9A shows the distribution of the Kendall rank correlation between the estimated subtype progression patterns and the ground truth for an increasing proportion of misdiagnosed subjects. As the proportion of misdiagnosed individuals increases the Kendall rank correlation decreases: the average Kendall rank correlation is 0.92 for $f_m = 0$, 0.91 for $f_m = 0.05$, 0.89 for $f_m = 0.1$, 0.88 for $f_m = 0.15$, and 0.83 for $f_m = 0.2$, still providing a good estimate of the subtype progression pattern for 15% and 20% outliers. Supplementary Figure 9B shows the distribution of the position of each event stage in the subtype progression patterns, i.e. the uncertainty in the subtype progression patterns estimated by SuStaIn, for increasing proportions of misdiagnosed subjects. As expected, the uncertainty increases as the proportion of misdiagnosed subjects increases, with an average standard deviation across simulated datasets of 0.07 for $f_m = 0$, 0.07 for $f_m = 0.05$, 0.08 for $f_m = 0.1$, 0.08 for $f_m = 0.15$, and 0.10 for $f_m = 0.2$.

Supplementary Figure 10A shows the distribution of the error in the proportion of subjects assigned to each cluster for increasing proportions of misdiagnosed subjects. SuStaIn accurately estimates the proportion of individuals belonging to each cluster: the mean absolute error is 0.026 for $f_m = 0$, 0.027 for $f_m = 0.05$, 0.038 for $f_m = 0.1$, 0.035 for $f_m = 0.15$, and 0.043 for $f_m = 0.2$. Supplementary Figure 10B shows the distribution of the proportion of individuals belonging to each cluster, i.e. the uncertainty in the proportion of individuals estimated by SuStaIn, for different numbers of subjects. As expected, this uncertainty increases slightly as the proportion of misdiagnosed individuals increases, with an average standard deviation across experiments of 0.027 for $f_m = 0$, 0.025 for $f_m = 0.05$, 0.030 for $f_m = 0.1$, 0.028 for $f_m = 0.15$, and 0.029 for $f_m = 0.2$. The distribution has a mean of zero for $f_m = 0$ and $f_m = 0.05$ subject experiments, indicating a non-biased estimate of the proportion of individuals belonging to each subtype for small proportions of misdiagnosed individuals. For $f_m = 0.1$, $f_m = 0.15$, and $f_m = 0.2$, SuStaIn slightly underestimates the proportion of individuals belonging to the largest cluster, and slightly overestimates the proportion of individuals belonging to the smallest cluster.

For increasing levels of misdiagnosed individuals, SuStaIn increasingly overestimates the number of subtypes in the dataset (the average number of estimated subtypes is 3.67 for $f_m = 0.05$, 4 for $f_m = 0.1$, 6 for $f_m = 0.15$, and 5.67 for $f_m = 0.2$ across datasets). However, each of the additional clusters is clearly an outlier cluster, accounting for a small number of individuals (most of the outlier clusters account for less than 5% of the data, and all account for less than 10%) and with no predominant progression pattern. This is somewhat to be expected as SuStaIn is specifically designed to group common features within a dataset and has no specific mechanism to model outliers, and is in contrast to the results we find in the ADNI (Figure 3) and GENFI studies (Figure 2), which indicate proportions of individuals of 27% for ADNI and 17% for GENFI belonging to the smallest subtypes. This supports the existence of distinct progression patterns.

Distinct and overlapping modes
Supplementary Figure 11A shows the distribution of the Kendall rank correlation between the estimated subtype progression patterns and the ground truth for distinct and overlapping modes. The Kendall rank correlation reduces when the modes are overlapping, as the SuStaIn model does not account for a spread of progression patterns. The average Kendall rank correlation is 0.94 for distinct modes and 0.81 for overlapping modes, still providing a reasonable correlation with the two ends of the

spectrum for overlapping modes. Inevitably the estimated clusters become closer together in the case of overlapping modes. This reduces the Kendall rank correlation between the estimated progression patterns and the ground truth progression pattern of each end of the spectrum. Supplementary Figure 11B shows the distribution of the position of each event stage in the subtype progression patterns, i.e. the uncertainty in the subtype progression patterns estimated by SuStaIn, for distinct and overlapping modes. This uncertainty has an average standard deviation across simulated datasets of 0.05 for distinct modes and 0.12 for overlapping modes. As expected the uncertainty in the position of the biomarker events for overlapping modes is asymmetric, because there is a tendency to choose two subtype progression patterns that are closer to the middle of the spectrum.

Supplementary Figure 12A shows the distribution of the error in the proportion of subjects assigned to each cluster distinct and overlapping modes: the mean absolute error is 0.032 for distinct modes and 0.067 for overlapping modes. Supplementary Figure 12B shows the distribution of the proportion of individuals belonging to each cluster, i.e. the uncertainty in the proportion of individuals estimated by SuStaIn, for distinct and overlapping modes, which has an average standard deviation across simulated datasets of 0.027 for distinct modes and 0.046 for overlapping modes. The distribution is centred around zero, i.e. the estimate of the proportion of individuals belonging to each cluster is unbiased for both distinct and overlapping modes.

For distinct modes SuStaIn estimates the correct number of clusters in all simulated datasets. For overlapping modes SuStaIn chooses two clusters for two datasets and three clusters for the remaining dataset. This demonstrates that SuStaIn may still recover distinct progression patterns when the data comes from a distributed set of progression patterns. However, SuStaIn still provides useful information about the extrema within the spectrum of progression patterns.

Supplementary Discussion

We assessed the effect of a variety of different properties in simulation: varying numbers of subjects, biomarkers and clusters, biomarker covariance, misdiagnosis and the effect of distinct vs. overlapping modes. The simulations demonstrate that SuStaIn shows good convergence properties across all different simulation settings. The results provide confidence in the ADNI and GENFI results by demonstrating that SuStaIn can provide accurate information on the subtype progression patterns for datasets with comparable numbers of subjects, biomarkers, and clusters to ADNI, and that whilst the subtype progression patterns are less accurate for datasets of the size of GENFI, SuStaIn still provides a meaningful indication of the progression in each subtype, showing a good correlation with the ground truth subtype progression patterns. Assessment of the effect of biomarker covariance indicates that the assumption of independent biomarker variance made by SuStaIn has little effect on the ability of SuStaIn to estimate the subtype progression patterns. The simulations further demonstrate that SuStaIn can handle a large (up to 20%) proportion of misdiagnosed individuals. Assessment of the performance of SuStaIn when the underlying progression patterns are made up of overlapping modes, which consist of a spectrum of progression patterns rather than multiple distinct progression patterns, demonstrates that SuStaIn may still recover distinct modes. However, SuStaIn is able to estimate the extrema of this space, providing a useful indication of the key modes of the spectrum of progression patterns.

Supplementary Figure 1

A.

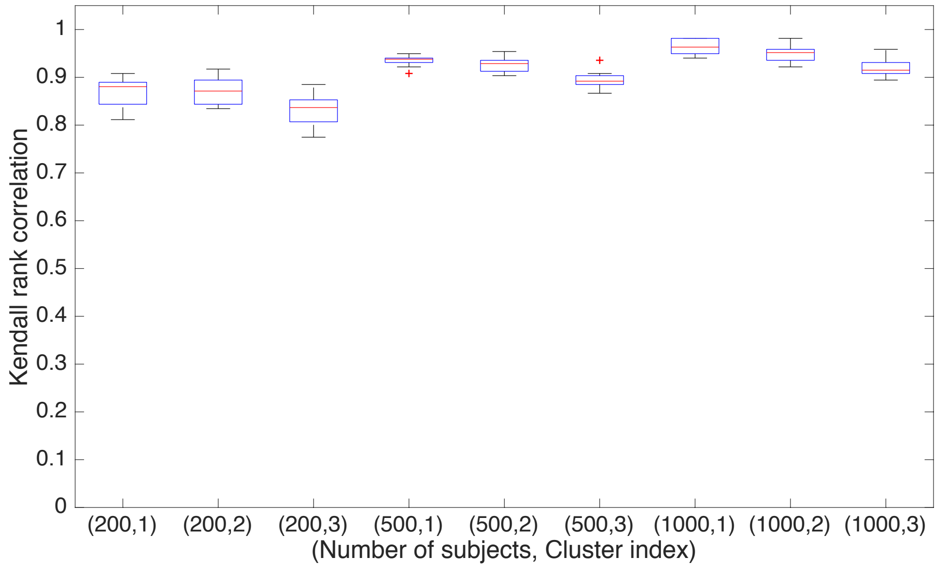

B.

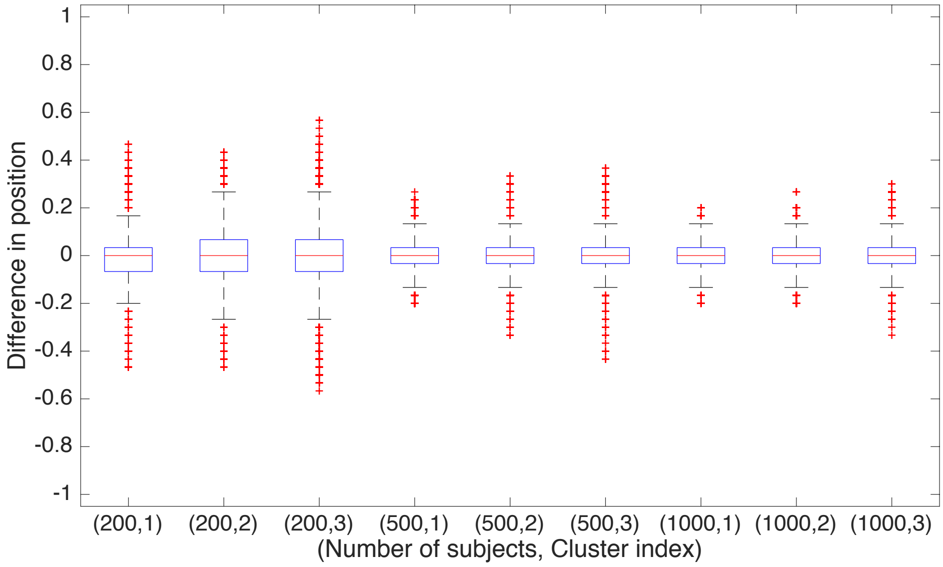

Effect of increasing subject numbers on the estimation of the subtype progression patterns by the SuStaIn algorithm. Subfigure A shows a box plot of the Kendall rank correlation between the most probable sequence estimated by SuStaIn and the ground truth across simulated datasets, with a higher Kendall rank correlation indicating more accurate estimation of the subtype progression patterns (maximum 1, minimum -1). Subfigure B shows a box plot of the distribution of the subtype progression patterns estimated by SuStaIn across simulated datasets, obtained from the MCMC samples of the uncertainty in the subtype progression patterns outputted by SuStaIn. In B., a wider distribution indicates a greater uncertainty estimate, and a distribution centred on zero indicates a non-biased estimate. The labels on the x-axis specify (population size, cluster index). Thus, within each group of the same population size the three boxes give an idea of how A and B change for the most prevalent subtype (cluster 1, $f = 4/9$) compared to the least prevalent subtype (cluster 3, $f = 2/9$).

Supplementary Figure 2

A.

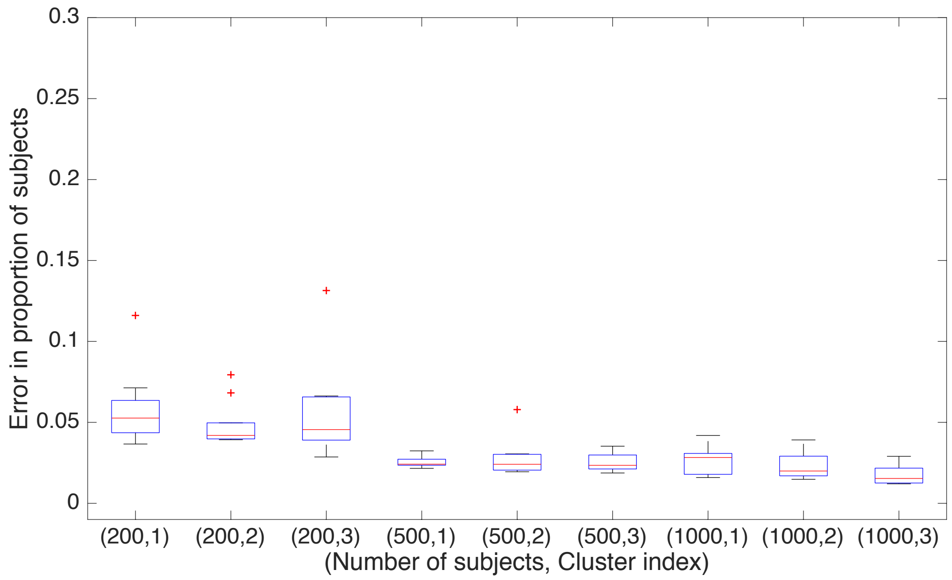

B.

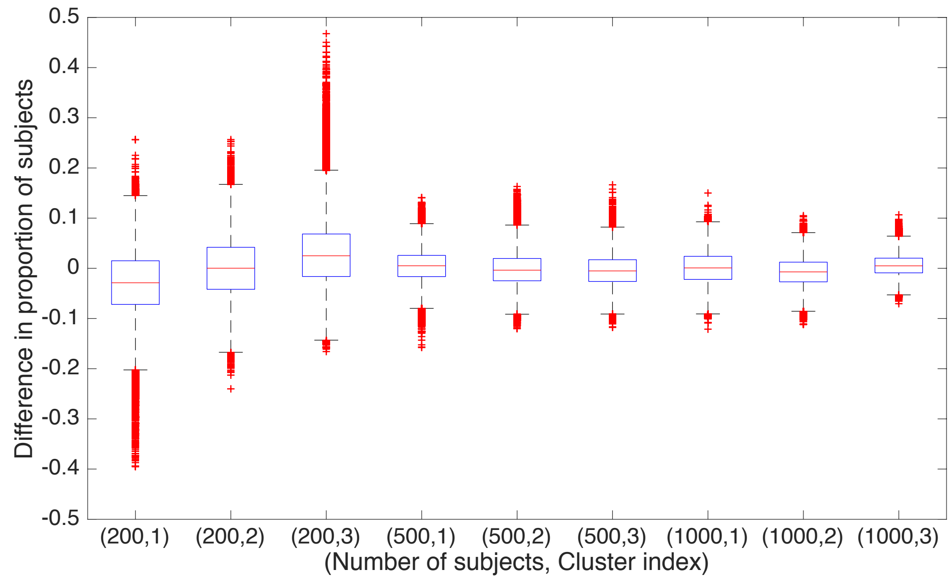

Effect of increasing subject numbers on the estimation of the proportion of individuals belonging to each subtype by the SuStaIn algorithm. Subfigure A shows a box plot of the mean absolute error between the most probable proportion estimated by SuStaIn and the ground truth across simulated datasets, with an error closer to zero indicating more accurate estimation of the subtype progression pattern. Subfigure B shows a box plot of the distribution of the proportion of individuals assigned to each subtype estimated by SuStaIn across simulated datasets, obtained from the MCMC samples of the uncertainty in the proportion of individuals belonging to each subtype outputted by SuStaIn. In B., a wider distribution indicates a greater uncertainty estimate and a distribution centred on zero indicates a non-biassed estimate, with a negative mean difference indicating underestimation and a positive mean difference indicating overestimation. The labels on the x-axis specify (population size, cluster index). Thus, within each group of the same population size the three boxes give an idea of how A and B change for the most prevalent subtype (cluster 1, $f = 4/9$) compared to the least prevalent subtype (cluster 3, $f = 2/9$).

Supplementary Figure 3

A.

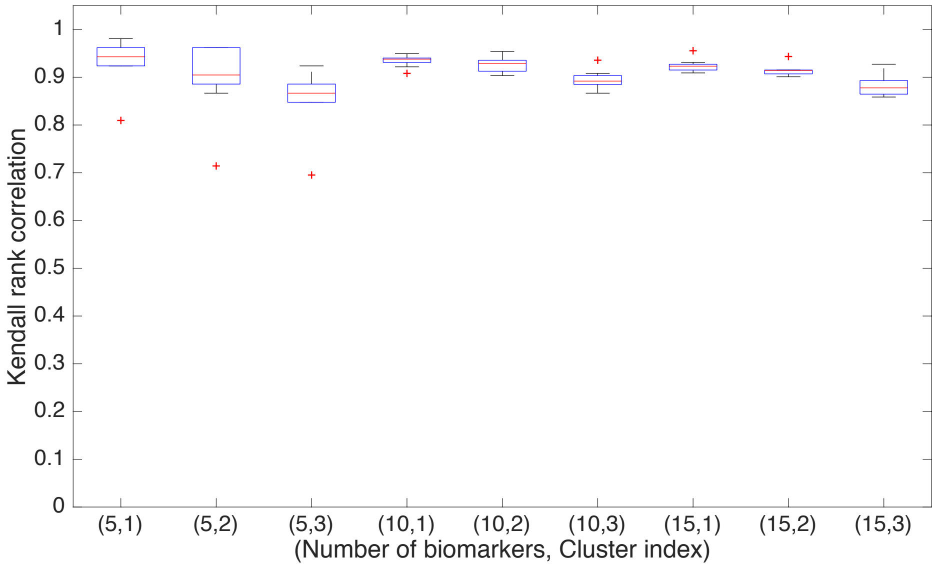

B.

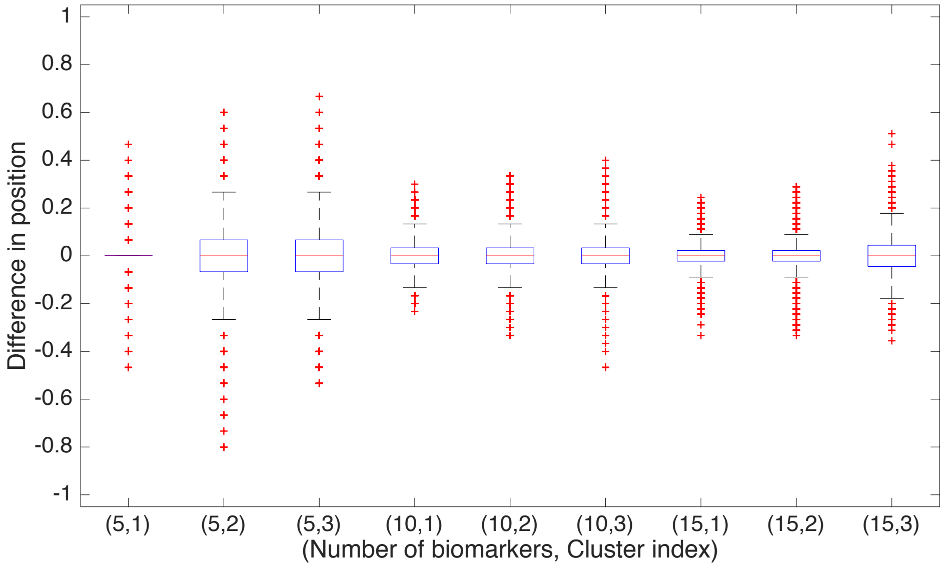

As Supplementary Figure 1, but for different numbers of biomarkers.

Supplementary Figure 4

A.

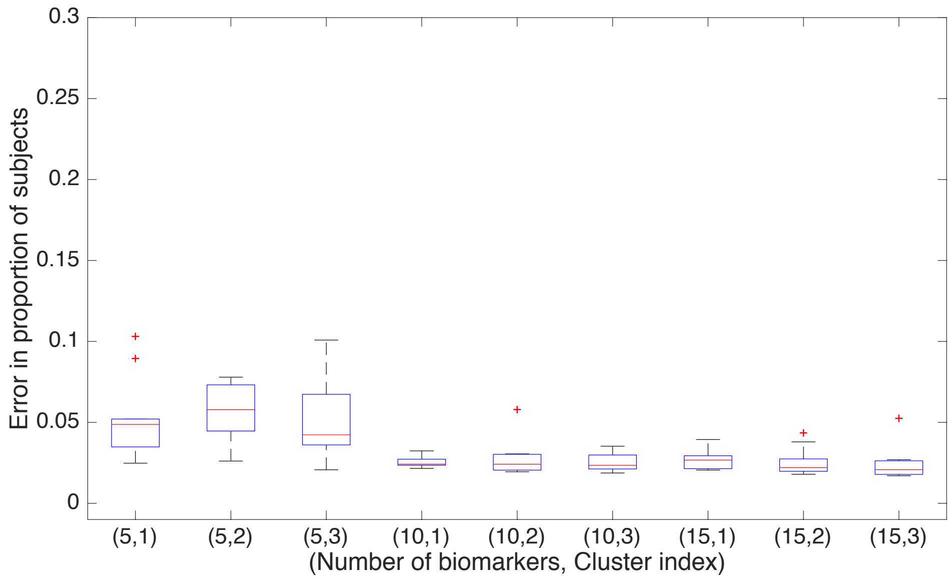

B.

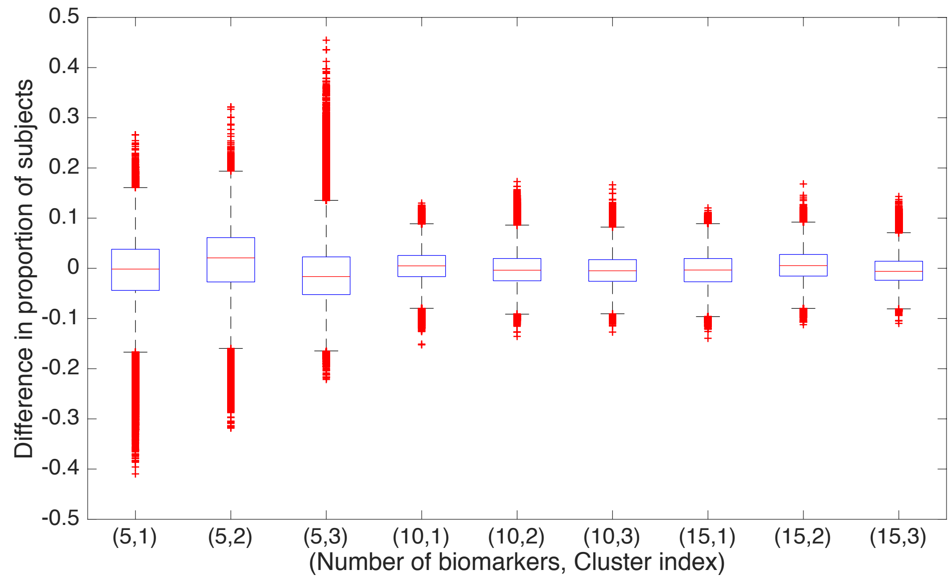

As Supplementary Figure 2, but for different numbers of biomarkers.

Supplementary Figure 5

A.

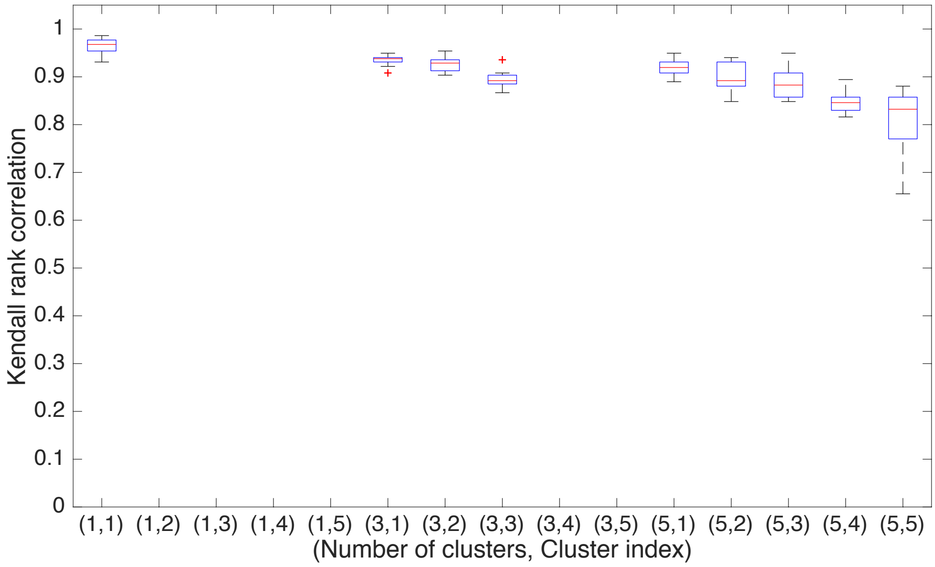

B.

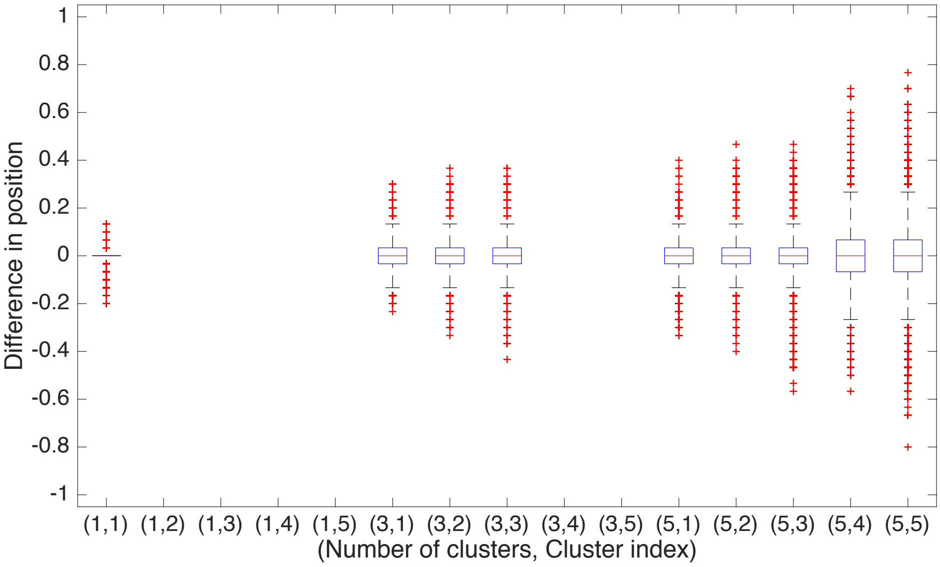

As Supplementary Figure 1, but for different numbers of clusters.

Supplementary Figure 6

A.

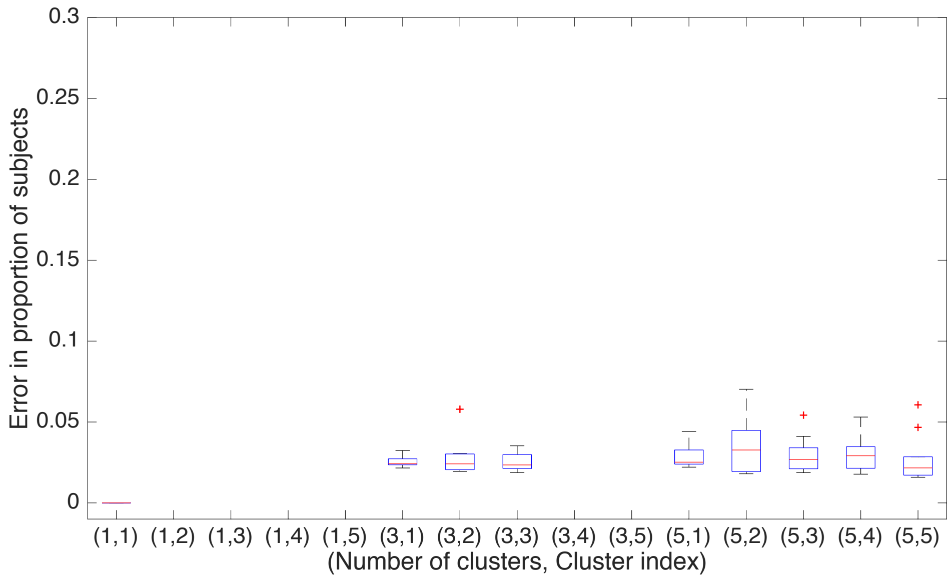

B.

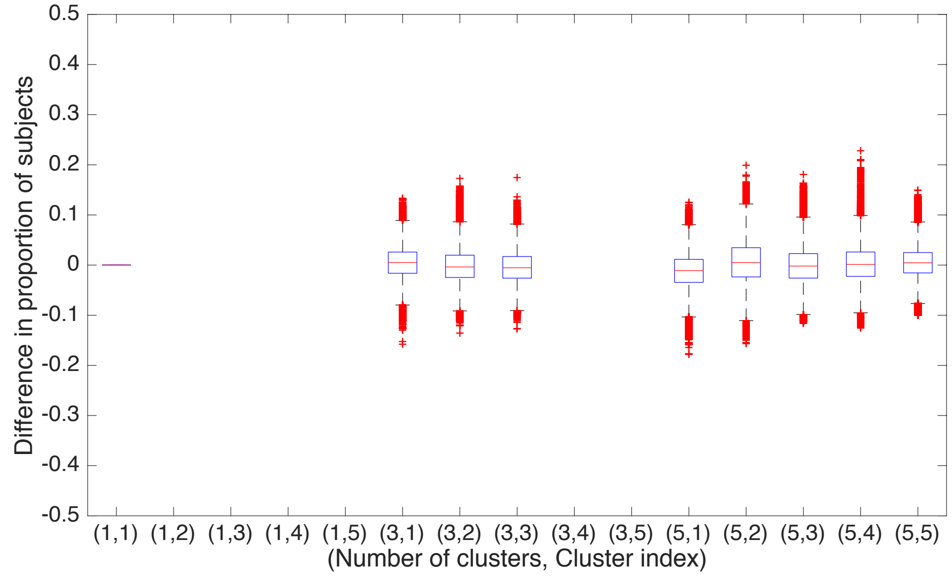

As Supplementary Figure 2, but for different numbers of clusters.

Supplementary Figure 7

A.

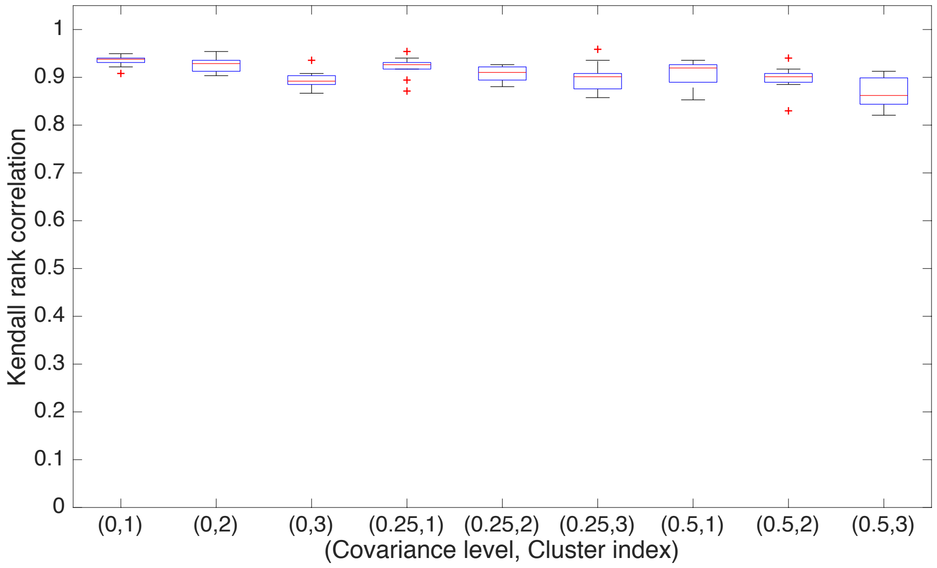

B.

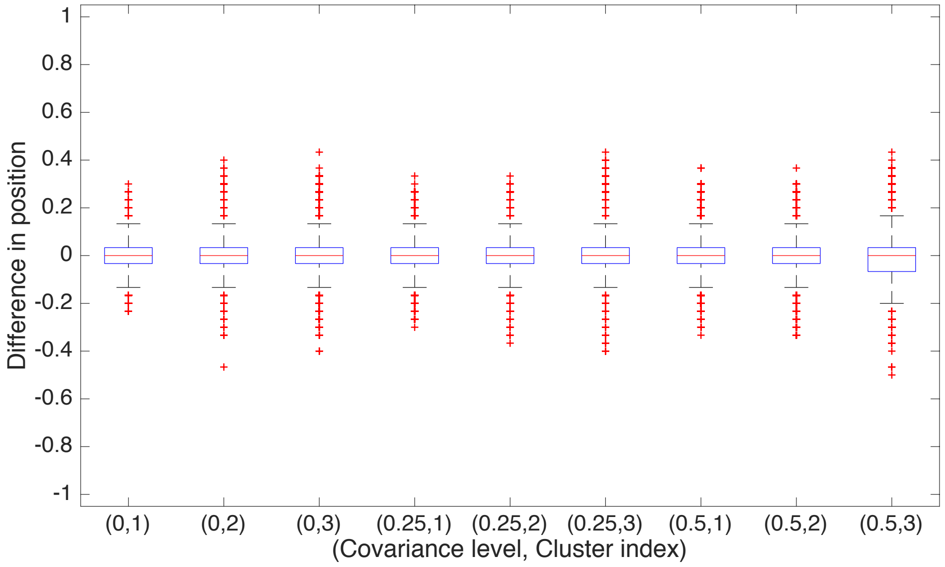

As Supplementary Figure 1, but for different biomarker covariance levels λ_{max} .

Supplementary Figure 8

A.

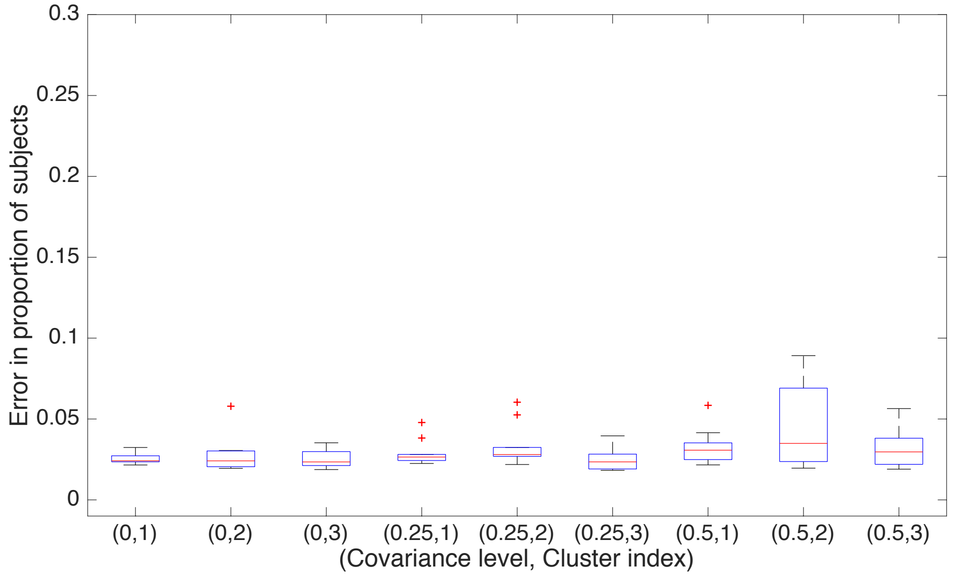

B.

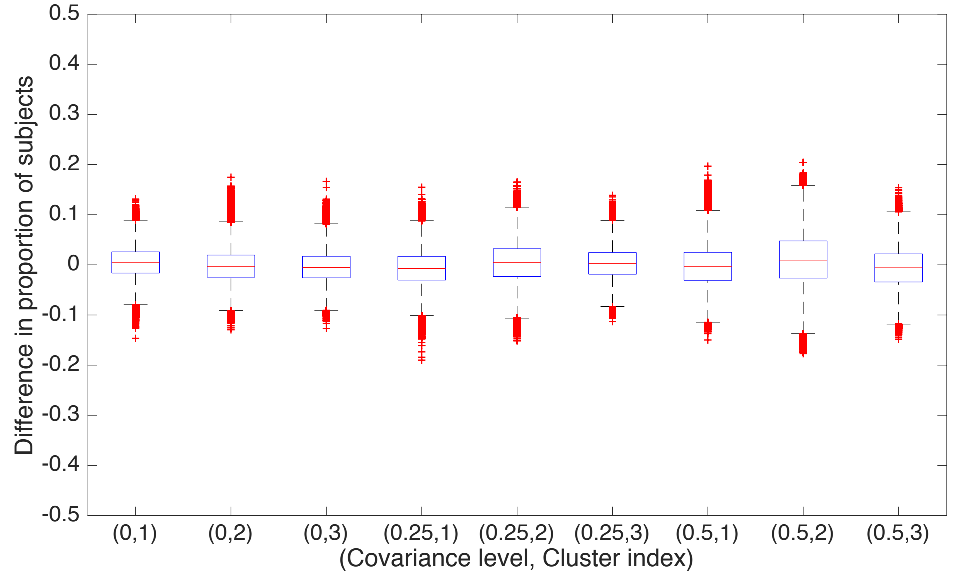

As Supplementary Figure 2, but for different biomarker covariance levels λ_{max} .

Supplementary Figure 9

A.

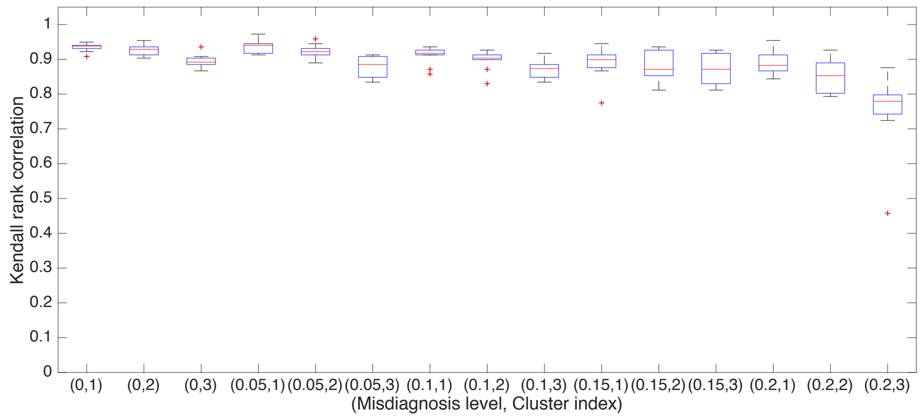

B.

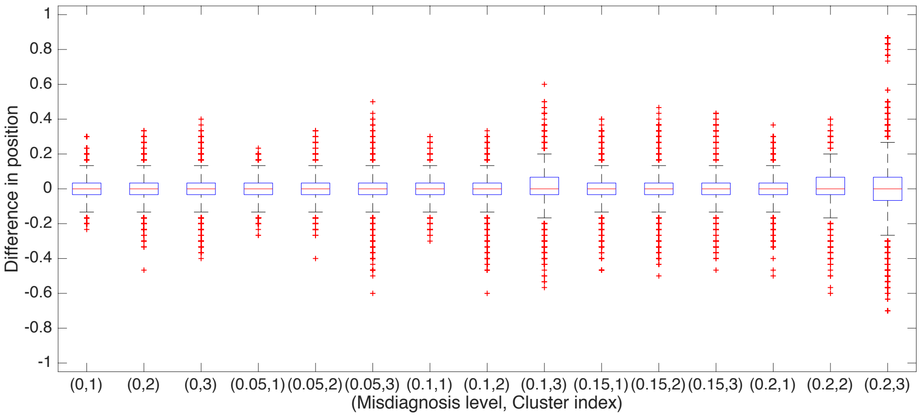

As Supplementary Figure 1, but for different proportions of misdiagnosed individuals.

Supplementary Figure 10

A.

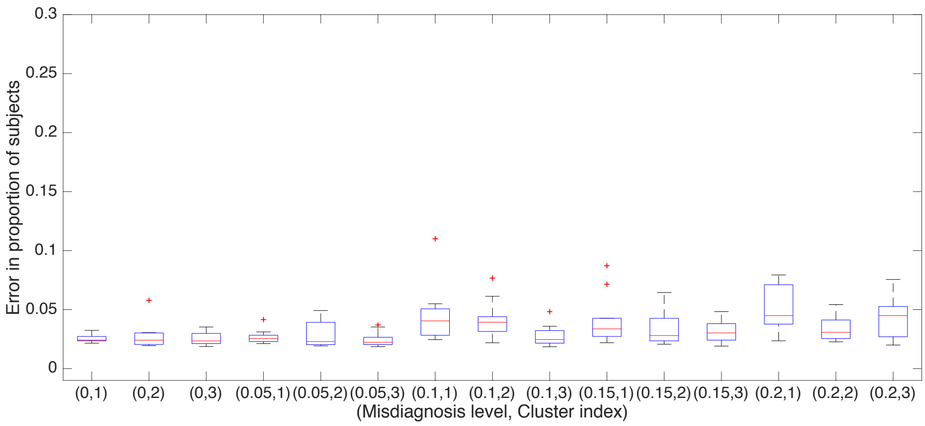

B.

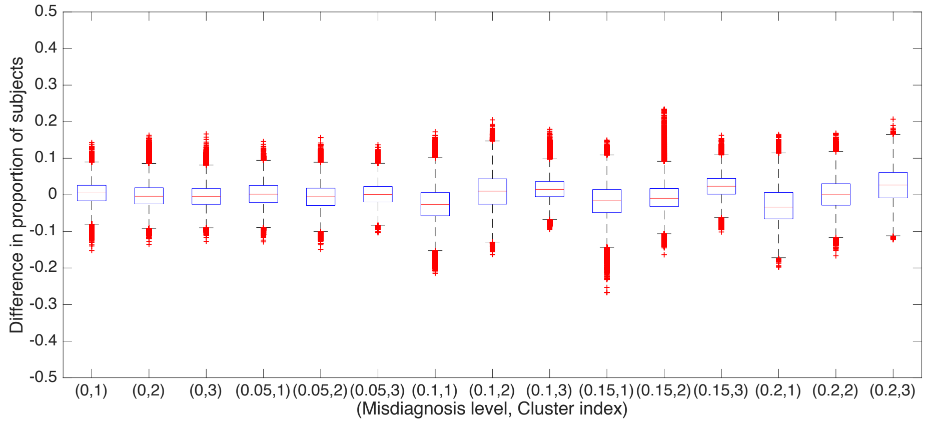

As Supplementary Figure 2, but for different proportions of misdiagnosed individuals.

Supplementary Figure 11

A.

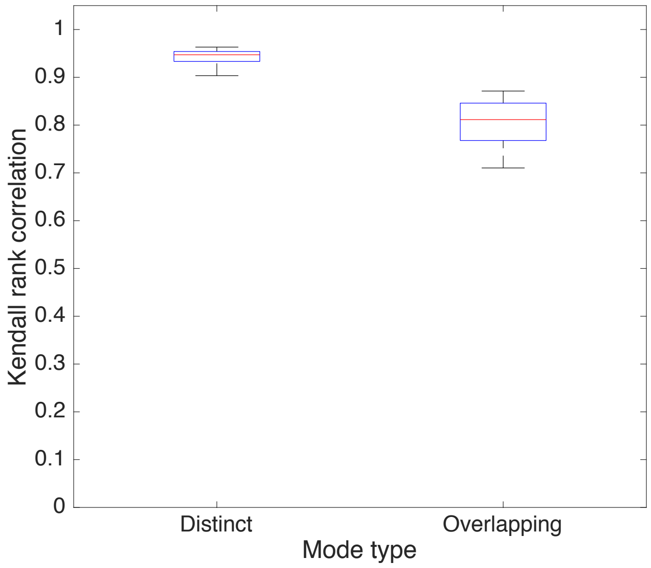

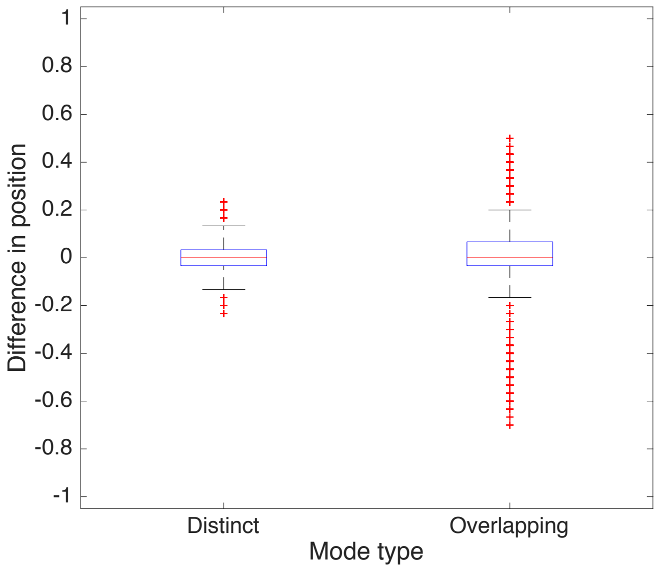

B.

As Supplementary Figure 1, but for different mode types.

Supplementary Figure 12

A.

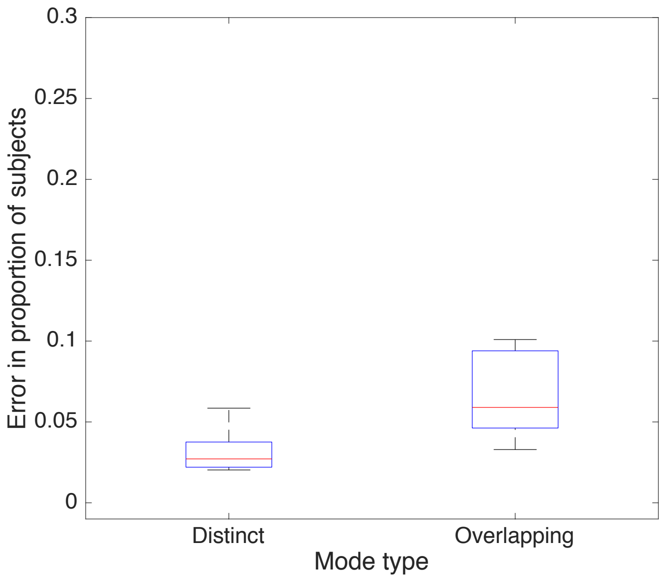

B.

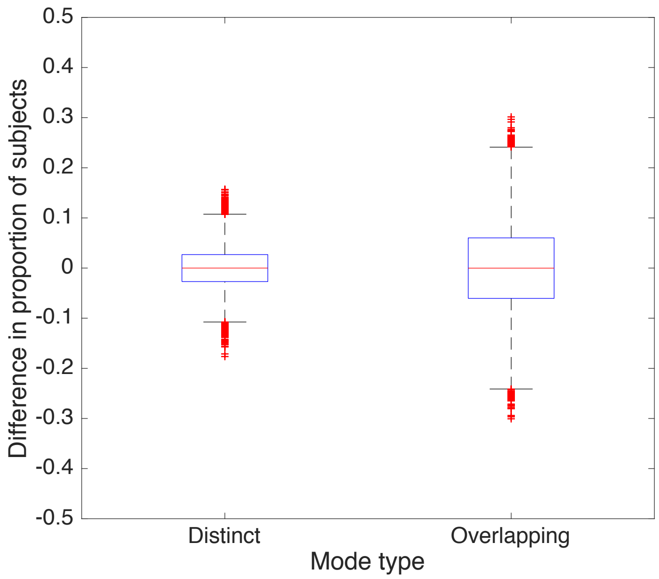

As Supplementary Figure 2, but for different mode types.

Supplementary Figure 13

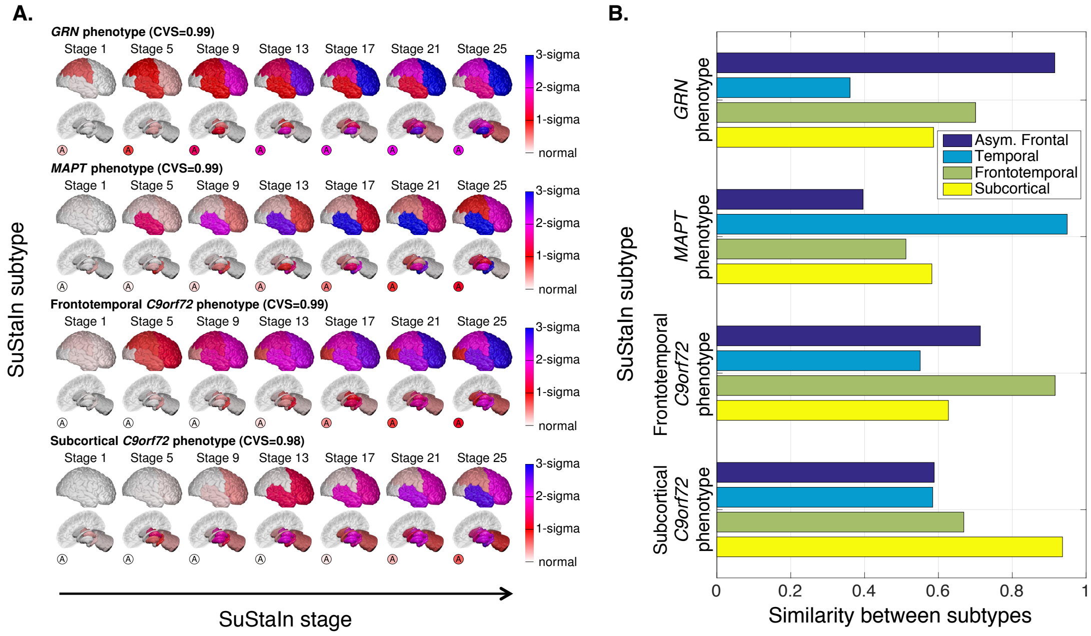

SuStaIn modelling of each genetic type in GENFI separately. Subfigure A shows the subtype progression patterns obtained from SuStaIn modelling of each genetic type. Diagrams structured as in Figure 2A. SuStaIn estimates four phenotypes in total: one phenotype in the *GRN* mutation carriers, which we describe as a *GRN* phenotype; one major phenotype in the *MAPT* mutation carriers (two groups: one major phenotype and a subsidiary group with no predominant progression pattern), which we describe as a *MAPT* phenotype; two phenotypes in the *C9orf72* mutation carriers, which we describe as a frontotemporal *C9orf72* phenotype and a subcortical *C9orf72* phenotype. Subfigure B shows the similarity (see Methods: Similarity between two progression patterns) between subtypes obtained from SuStaIn modelling of each genetic type in GENFI separately (shown in A) and subtypes obtained from SuStaIn modelling of all mutation carriers in GENFI as a whole (shown in Figure 2A). Subfigure B shows that the subtype progression patterns obtained from SuStaIn for each genetic type in GENFI separately are in good agreement with the subtype progression patterns estimated from pooling all mutation carriers in GENFI (Figure 2A). This result demonstrates that SuStaIn is able to correctly identify the *GRN* and *MAPT* neuroanatomical phenotypes without any knowledge of genotype, and provides further evidence that the *C9orf72* group is best described as two distinct neuroanatomical phenotypes.

Supplementary Figure 14

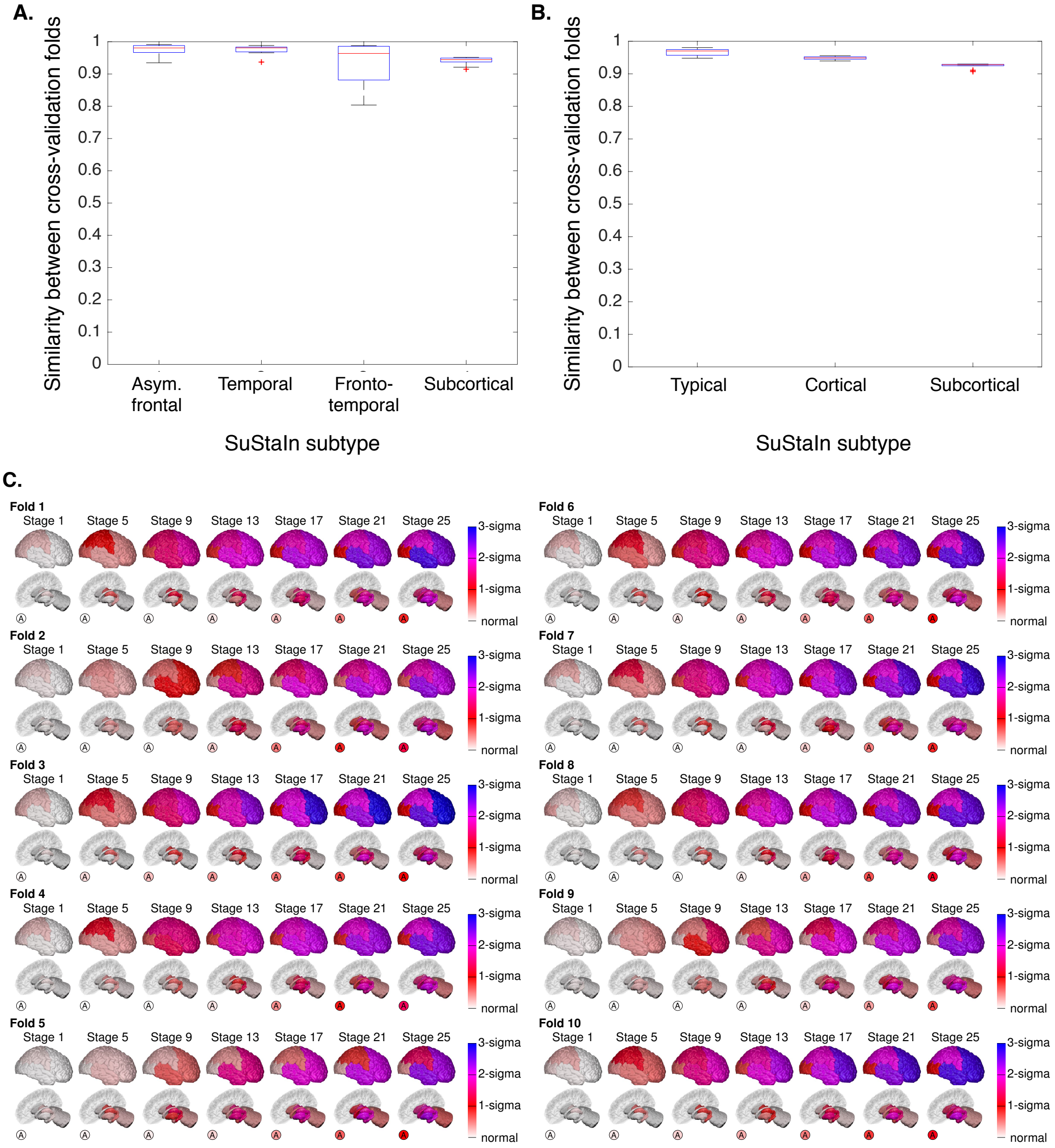

Reproducibility of the GENFI and ADNI results under cross-validation. Subfigure A shows the reproducibility of the GENFI results in Figure 2A and subfigure B shows the reproducibility of the ADNI results in Figure 3. Each box plot shows the similarity between the SuStaIn subtypes estimated from each pair of cross-validation folds for A. the GENFI dataset and B. the ADNI dataset. Subfigure C shows the variation across folds for an example progression pattern: the frontotemporal lobe subtype estimated in GENFI. This example subtype was chosen to demonstrate the maximal variability of a subtype progression pasttern across cross-validation folds.

Supplementary Table 1

	GRN	*MAPT*	*C9orf72*
Asymmetric frontal	93% (13)	9% (1)	17% (4)
Temporal	0% (0)	91% (10)	25% (6)
Frontotemporal	0% (0)	0% (0)	38% (9)
Subcortical	7% (1)	0% (0)	21% (5)
Accuracy	93% (13/14)	91% (10/11)	58% (14/24)

As Table 1, but using a simple (non-optimised) subtype assignment strategy. The simple assignment strategy (see Methods: Classification of mutation groups using subtypes) only modestly reduces the accuracy of the assignment, giving a balanced accuracy of 81% rather than 86%. Again, the results show that SuStaIn can accurately discriminate genotypes, validating the ability of SuStaIn to identify distinct phenotypes that align with known genetic groups.

Supplementary Table 2

	GRN	*MAPT*	*C9orf72*
Severe frontal	64% (9)	9% (1)	17% (4)
Severe temporal	0% (0)	64% (7)	17% (4)
Mild frontotemporal	36% (5)	27% (3)	67% (16)
Accuracy	64% (9/14)	64% (7/11)	67% (16/24)

As Table 2, but using a simple (non-optimised) subtype assignment strategy for comparison with Supplementary Table 1. Again, the results show that SuStaIn (Supplementary Table 1) provides much better discrimination of the different genotypes than the subtypes-only model shown here, demonstrating the added utility of a model that accounts for heterogeneity in disease stage.

Supplementary Table 3

	Likelihood ratio	Likelihood ratio test p-value
SuStaIn vs. Subtypes-only model	4.65×10^{3}	3.96×10^{-5}
SuStaIn vs. Stages-only model	3.05×10^{2}	7.18×10^{-4}

Comparison of SuStaIn for predicting the risk of conversion from mild cognitive impairment to Alzheimer's disease with models that only account for heterogeneity in either disease subtype (subtypes-only model) or disease stage (stages-only model). The likelihood ratio of SuStaIn vs. alternative model is the ratio of the likelihood of a Cox Proportional Hazards Model estimated using subtypes and stage from SuStaIn as predictors compared to the likelihood of a Cox Proportional Hazards Model estimated using variables from the alternative model as predictors. A likelihood ratio of above 1 indicates that the predictors from SuStaIn provide a better fit. The likelihood ratio test p-value indicates whether the predictors from SuStaIn provide a significantly better fit than the alternative model predictors. This result shows that SuStaIn provides a significantly better estimate of the risk of conversion from mild cognitive impairment to Alzheimer's disease than a subtypes-only or stages-only model, i.e. both the disease subtypes and the disease stages estimated by SuStaIn are useful for estimating the risk of conversion.

Supplementary Figure 15

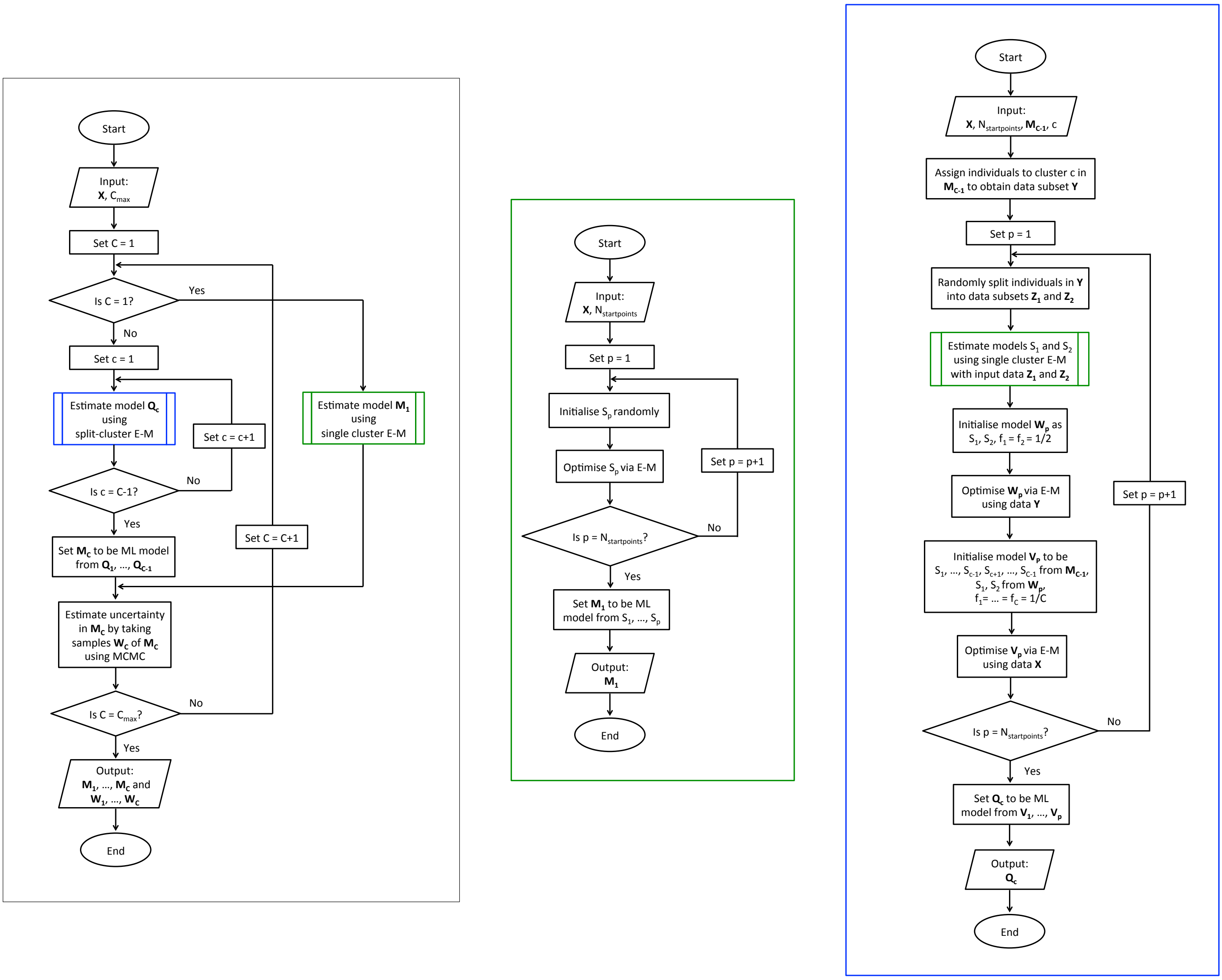

Flowchart of the processes involved in the SuStaIn model fitting. The overall SuStaIn model fitting procedure is shown in the black box, with the sub-procedures single cluster E-M and split-cluster E-M being shown in the green and blue boxes, respectively. $\mathbf{M}_C$ denotes a SuStaIn model with C clusters, consisting of a set of sequences $\boldsymbol{S}_1, \dots, \boldsymbol{S}_C$ and fractions $f_1, \dots, f_C$.

Supplementary Table 4

Biomarker type	Dataset(s)	Individual biomarkers	Use
Regional MRI volumes	GENFI<br>ADNI 3T<br>ADNI 1.5T	Frontal lobe	To estimate the SuStaIn models in Figures 2A, 3, 4 and Supplementary Figure 13A (following correction for head size, demographics, and number of *APOE4* alleles (ADNI only), and transformation to a positive z-score relative to a control population)
		Temporal lobe	
		Parietal lobe	
		Occipital lobe	
		Cingulate	
		Insula	
		Accumbens	
		Amygdala	
		Caudate	
		Hippocampus	
		Pallidum	
		Putamen	
		Thalamus	
	GENFI	Cerebellum	
		Asymmetry	
Head size	GENFI<br>ADNI 3T<br>ADNI 1.5T	Total intracranial volume	To correct regional MRI volumes for head size (correction learnt in control population and propagated to whole population)
Genotype	GENFI	Non-carrier	To define a control population
		Mutation carrier	To define the population in which to estimate the SuStaIn model in Figure 2A
		Mutation type: *GRN*, *C9orf72* or *MAPT*	To define the population in which to estimate the SuStaIn models in Supplementary Figure 13A;<br>To compare the SuStaIn model in Figure 2A to the genotype labels (Figure 2B, Figure 5A, Table 1)
	ADNI 3T<br>ADNI 1.5T	Number of *APOE4* alleles	To correct regional MRI volumes for number of *APOE4* alleles (correction learnt in control population and propagated to whole population)
Diagnosis	GENFI	Pre-symptomatic or symptomatic	To look at the identifiability of SuStaIn subtypes (Figure 5A and Figure 5B): To look at the distribution of the SuStaIn stages amongst diagnostic groups (Figure 5C and Figure 5D)
	ADNI 3T<br>ADNI 1.5T	Diagnosis label: CN, MCI or AD	
		CN	To define a control population (together with an amyloid negative CSF result)
CSF Abeta1-42	ADNI 3T<br>ADNI 1.5T	Amyloid negative (CSF Abeta1-42 > 192pg per ml)	To define a control population (together with a diagnosis of cognitively normal)
Demographic information	GENFI<br>ADNI 3T<br>ADNI 1.5T	Age	To correct regional MRI volumes for subject demographics (correction learnt in control population and propagated to whole population)
		Sex	
		Years of Education	

Table detailing the biomarkers used in the SuStaIn modelling for different datasets. CN = cognitively normal; MCI = mild cognitive impairment, AD = Alzheimer's disease; CSF = cerebrospinal fluid.

Supplementary Table 5

Dataset	Subset	J	I	C_{max}	N
GENFI	All mutation carriers	172	15	5	33
	GRN	76		3	
	C9orf72	63			
	MAPT	33			
ADNI	3T	793	13	5	31
	1.5T	576			30

Table detailing the SuStaIn algorithm settings for different data subsets. J is the number of subjects, I is the number of biomarkers, $C_{\max}$ is the maximum number of clusters fitted, and N is the number of z-score events.

Supplementary Table 6

	GENFI		ADNI 3T		ADNI 1.5T	
	R	z_{max}	R	z_{max}	R	z_{max}
Frontal	3	5	2	3	3	5
Temporal	3	5	3	5	3	5
Parietal	2	3	3	5	3	5
Occipital	1	2	2	3	2	3
Cingulate	2	3	3	5	2	3
Insula	3	5	2	3	2	3
Accumbens	3	5	3	5	2	3
Amygdala	3	5	3	5	3	5
Caudate	1	2	1	2	1	2
Hippocampus	3	5	3	5	3	5
Pallidum	1	2	2	3	2	3
Putamen	3	5	2	3	2	3
Thalamus	2	3	2	3	2	3
Cerebellar	1	2				
Asymmetry	2	3				

Table detailing the SuStaIn algorithm settings for different biomarkers for the GENFI, ADNI 3T and ADNI 1.5T datasets. The first column on left hand side lists the biomarkers included in the SuStaIn model. R is the number of z-scores included for biomarker i, and $z_{\max}$ is the maximum z-score modelled for biomarker i.
